# Low Quality of Warfarin Therapy is Associated With Female Gender but Not With Polypharmacy in Patients With Atrial Fibrillation

**DOI:** 10.3389/fphar.2021.651799

**Published:** 2021-04-26

**Authors:** Kojiro Takamoto, Jun-ichi Sakamoto, Satoyasu Ito, Takeshi Kimura, Eri Manabe, Toshiyuki Shikata, Masanori Asakura, Masaharu Ishihara, Takeshi Tsujino

**Affiliations:** ^1^ Graduate School of Pharmacy, Hyogo University of Health Sciences, Kobe, Japan; ^2^ Hyogo College of Medicine Sasayama Medical Center, Sasayama, Japan; ^3^ Department of Pharmacy, Hyogo College of Medicine Hospital, Nishinomiya, Japan; ^4^ Department of Pharmacy, School of Pharmacy, Hyogo University of Health Sciences, Kobe, Japan; ^5^ Department of Cardiovascular and Renal Medicine, Hyogo College of Medicine, Nishinomiya, Japan

**Keywords:** polypharmacy, atrial fibrillation, warfarin, PINRR, TTR, female gender, sex differences

## Abstract

**Background:** We examined the impact of polypharmacy on the quality of the anticoagulation therapy in patients with atrial fibrillation. We also examined the factors that affect the stability of warfarin therapy.

**Methods and Results:** This retrospective study was conducted using data from 157 consecutive outpatients with atrial fibrillation in a single tertiary referral hospital. Patients who were prescribed warfarin continuously and for whom PT-INR was examined at least three times in a year were included in this study. We examined the quality of warfarin therapy using time in the therapeutic INR range (TTR), percentage of PT-INR determinations in range (PINRR), and the coefficient variation (CV) of PT-INR. We found that the number of prescribed medicines was significantly associated with high BMI and low eGFR, but not with TTR, PINRR, and the coefficient variation of PT-INR in patients with atrial fibrillation. We also found that female gender was independently associated with low PINRR in this study population.

**Conclusion:** Polypharmacy did not deteriorate the quality of warfarin therapy in patients with atrial fibrillation treated in the tertiary referral hospital. Female gender was an independent predictor of the low quality of warfarin therapy.

## Introduction

Declining fertility rates and increasing life expectancy has led to a rapid increase in the average age of the population; in addition, the incidence of atrial fibrillation (AF) is rapidly increasing in the elderly population (Ohsawa et al., 2005; Inoue et al., 2009). AF is a common arrhythmia and strong risk factor for cardiogenic thromboembolism (Wolf et al., 1991; Feinberg et al., 1995). Appropriate treatment including anticoagulant medication, is needed to prevent cardiogenic thromboembolism (Hart et al., 1998; Connolly et al., 2009; Hori et al., 2012; Kim et al., 2018; Cheng et al., 2019). In the past, the vitamin K inhibitor warfarin, was the only orally active anticoagulant for prevention of stroke in patients with AF in Japan. However, in recent years, direct oral anticoagulants (DOACs), such as dabigatran, edoxaban, rivaroxaban, and apixaban, have been used widely. DOACs have decreased the risk of cardiogenic thromboembolic events and the mortality of patients with AF (Halperin et al., 2014; Halvorsen et al., 2014; Toda Kato et al., 2016; Mandy et al., 2017). However, warfarin is still widely used because DOACs are more expensive than warfarin and cannot be used in patients with impaired renal function. Warfarin is known to interact with many medications, and the effects are different in each individual. It is well known that strict control of the international normalized ratio of prothrombin time (PT-INR) during warfarin therapy is required to ensure not only efficacy but also safety. However, in previous meta-analyses, the quality of anticoagulation control with warfarin was proven to be poor, with an estimated time in the therapeutic INR range (TTR) between 55 and 64% (van Walraven et al., 2006; Wan et al., 2008; Baker et al., 2009; Cios et al., 2009). Thus, warfarin administration should be refined for the safe and effective treatment of AF patients.

The use of multiple medications, widely referred to as polypharmacy, is a clinical issue in the older population (Hajjar et al., 2007). There is no standard cut-off point with regard to multiple medications, but polypharmacy is usually considered as five or more medications in Japan, in accordance with the Guidelines for Medical Treatment and its Safety in the Elderly 2015 (The Japan Geriatrics Society, 2015). Polypharmacy has been shown to impair adherence to medication. Moreover, the potency of warfarin is known to be affected by concomitant medications. Therefore, polypharmacy could deteriorate the quality of warfarin therapy. In fact, polypharmacy has been shown to be associated with mortality and bleeding complications in patients with AF (Abdelhafiz and Wheeldon, 2008; Proietti et al., 2016). However, it is not fully elucidated whether polypharmacy worsens warfarin control. The examination of the impact of polypharmacy on the stability of warfarin therapy is important for the safe use of warfarin. In this study, we examined how polypharmacy affected the stability of warfarin therapy in patients with AF. We also examined other factors affecting warfarin control.

## Methods

### Population and Data Collection

This retrospective study was conducted using the data from outpatients with AF visiting the Department of Cardiovascular and Renal Medicine, Hyogo College of Medicine Hospital between April 2013 and March 2014. Patients who were prescribed warfarin continuously and received PT-INR examination at least three times during this period were included in the study. Patients who were admitted to the hospital during this period were excluded. We collected data from their clinical records, including demographics, comorbidity, and medications. Polypharmacy was defined to be prescribed five or more medications regularly in this study period. We used the TTR, the percentage of PT-INR in the therapeutic range (PINRR), and coefficient variation (CV) of PT-INR as index to evaluate the stability of warfarin therapy. TTR was determined using a linear interpolation method between consecutive PT-INR values in each patient as reported by the Rosendaal method (Rosendaal et al., 1993). Japanese Guidelines for Pharmacotherapy of Atrial Fibrillation recommended that PT-INR should be controlled between 2.0 and 3.0 in patients of <70 years of age and between 1.6 and 2.6 in those of ≥70 years of age between April 2013 and March 2014 (The Japan Circulation Society, 2014). However, real-world data revealed that PT-INR values in patients of <70 years were in the same range as those in patients of ≥70 years of age in Japan (Okumura et al., 2011; Tomita et al., 2013). JCS/JHRS 2020 Guideline on Pharmacotherapy of Cardiac Arrhythmias now recommends that PT-INR should be controlled between 1.6 and 2.6 regardless of age for primary prevention (The Japan Circulation Society/Japanese Heart Rhythm Society Joint Guidelines et al., 2020). Thus, we determined that PT-INR levels were in the therapeutic range when they were between 1.6 and 2.6.

Based on comorbidity information, we calculated each patient’s stroke risk predictor score (CHADS2, and CHA2DS2-VASc score) and bleeding risk predictor score (HAS-BLED score) in accordance with previous reports (Gage et al., 2001; Lip et al., 2010; Pisters et al., 2010). We calculate estimated glomerular filtration rate (eGFR) from the Japanese equation: eGFR (ml/min/1.73 m^2^) = 194 × serum creatinine −1.094 × age −0.287 (×0.739 if female). Hypertension was defined as a systolic blood pressure of ≥140 mmHg, a diastolic blood pressure of ≥90 mmHg, or the use of anti-hypertensive medication. Diabetes mellitus was defined as fasting plasma glucose of ≥126 mg/dl, HbA1c of ≥6.5%, or the use of anti-diabetic medication. Dyslipidemia was defined as low-density lipoprotein cholesterol (LDL-C) of ≥140 mg/dl, high-density lipoprotein cholesterol (HDL-C) of ≤40 mg/dl, and triglycerides (TG) of ≥150 mg/dl, or the use of anti-dyslipidemia medication. This investigation was approved by the Ethics Committee of Hyogo College of Medicine Hospital (#1900) and the Ethics Committee of Hyogo University of Health Sciences (#15009). All analyses were performed in accordance with approved guidelines and regulations. Because this study is a non-invasive retrospective observational study, informed consent of patients was obtained in the opt-out way by disclosing information of the study.

### Statistical Analysis

All statistical analyses were performed with EZR on R Commander Version 1.35 (Saitama Medical Center, Jichi Medical University, Saitama, Japan) (Kanda, 2013), which is a graphical user interface for R (The R Foundation for Statistical Computing, Vienna, Austria). The data were presented as the mean ± standard deviation (SD) for normally distributed variables, the median (interquartile range) for not normally distributed variables, or the number (percentage) for categorical variables. The differences in categorical variables between groups were assessed using Fisher’s exact test. The differences in continuous variables between groups were assessed using unpaired Student’s *t*-test for normally distributed variables, or Mann-Whitney’s *U*-test for not normally distributed variables. With regard to continuous variables, the correlation between each variable was examined using Spearman’s rank correlation coefficient. The factors that were independently associated with the number of drugs were analyzed using linear regression analysis. Factors with *p* < 0.10 in Spearman’s rank correlation coefficient were selected as independent factors in the multivariate analysis.

## Results

### Study Population

This retrospective study was conducted using data from 157 outpatients with non-valvular AF. The characteristics of the study population are shown in Table 1. The median of the number of prescribed drugs in the whole study population was seven, and 125 patients (79.6%) were using five or more prescription drugs regularly. The average age of the study population was 72.1 ± 8.7 years of age and there were 121 male patients (77.1%). The number of patients with CHADS2 score more than or equal to 2 was 93 (59.2%). The number of patients with CHA2DS2-VASc score more than or equal to 2 was 119 (75.8%). The number of patients with HAS-BLED score more than or equal to 3 was 7 (4.5%). We evaluated the quality of warfarin therapy with TTR and PINRR. The median TTR was 84% and the median PINRR was 78%. The mean ± SD TTR was 79.5 ± 20.7%, which was comparable with previous reports from Japan (Okumura et al., 2011; Tomita et al., 2013). PT-INR values in patients of <70 years of age were almost the same as those in patients of ≥70 years of age in our study population (<70 years of age vs. ≥70 years of age; 2.03 ± 0.27 vs. 1.99 ± 0.27, *p* = 0.371). Thus, it was reasonable to calculate PINRR as the percentage of PT-INR between 1.6 and 2.6 in all patients as previously reported in Japan (Okumura et al., 2011; Tomita et al., 2013). Medications potentiating the action of warfarin were prescribed in 90 patients (57.3%), and medications weakening the effects of warfarin (mostly glucocorticoid formulations) were prescribed in nine patients (5.7%).

**TABLE 1:** Clinical characteristics.

	All			
		NP	PP	*p*-value
*N*	157	32	125	
Age (years)	72.1 ± 8.7	73.7 ± 8.3	71.7 ± 8.7	0.368
BMI (kg/m^2^)	23.6 ± 3.7	22.5 ± 4.4	23.9 ± 3.5	0.144
Male	121 (77.1)	24 (75.0)	97 (77.6)	0.814
Paroxysmal AF	60 (38.2)	12 (37.5)	48 (38.4)	1.000
Comorbid diseases				
Heart failure	51 (32.5)	4 (12.5)	47 (37.6)	0.006
Hypertension	115 (73.2)	19 (59.4)	96 (76.8)	0.072
Diabetes mellitus	45 (28.7)	4 (12.5)	41 (32.8)	0.028
Dyslipidemia	54 (34.4)	11 (34.4)	43 (34.4)	1.000
Stroke	14 (8.9)	2 (6.3)	12 (9.6)	0.737
Ischemic heart disease	32 (20.4)	2 (6.3)	30 (24.0)	0.027
Peripheral artery disease	5 (3.2)	0 (0)	5 (4.0)	0.584
Vascular disease	35 (22.3)	2 (6.3)	33 (26.4)	0.016
Abnormal renal function	1 (0.6)	0 (0)	1 (0.8)	1.000
Abnormal liver function	3 (1.9)	0 (0)	3 (2.4)	1.000
Bleeding	36 (22.9)	8 (25.0)	28 (22.4)	0.814
Laboratory test				
WBC (×10^2^/µl)	57.2 ± 15.8	59.6 ± 20.5	56.5 ± 14.4	0.345
RBC (×10^4^/µl)	442 ± 56	449 ± 53	400 ± 57	0.483
Hb (g/dl)	13.7 ± 1.7	14.0 ± 1.6	13.6 ± 1.7	0.220
Ht (%)	41.1 ± 4.7	42.0 ± 4.4	40.9 ± 4.8	0.227
Plt (×10^4^/µl)	17.6 ± 5.2	18.6 ± 5.4	17.4 ± 5.2	0.269
Cr (mg/dl)	1.0 ± 0.3	0.9 ± 0.2	1.0 ± 0.4	0.021
eGFR (ml/min/1.73m^2^)	60.7 ± 18.4	67.3 ± 18.0	59.1 ± 18.2	0.028
AST (U/L)	24.2 ± 8.6	24.3 ± 7.9	24.1 ± 8.7	0.905
ALT (U/L)	18.7 ± 7.8	19.0 ± 7.3	18.6 ± 8.0	0.796
CHADS_2_ score	2 (1, 3)	1 (1, 2)	2 (1, 3)	0.002
0	15 (9.6)	5 (15.6)	10 (8.0)	
1	49 (31.2)	14 (43.8)	35 (28.0)	
2	44 (28.0)	10 (31.3)	34 (27.2)	
3	35 (22.3)	3 (9.4)	32 (25.6)	
4	9 (5.7)	0 (0)	9 (7.2)	
5	3 (1.9)	0 (0)	3 (2.4)	
6	2 (1.3)	0 (0)	2 (1.6)	
CHA_2_DS_2_-VASc score	2 (2, 4)	2 (1.8, 3)	3 (2, 4)	0.133
0	6 (3.8)	1 (3.1)	5 (4.0)	
1	32 (20.4)	7 (21.9)	25 (20.0)	
2	43 (27.4)	13 (40.6)	30 (24.0)	
3	26 (16.6)	4 (12.5)	22 (17.6)	
4	28 (17.8)	6 (18.8)	22 (17.6)	
5	13 (8.3)	1 (3.1)	12 (9.6)	
6	6 (3.8)	0 (0)	6 (4.8)	
7	2 (1.3)	0 (0)	2 (1.6)	
8	1 (0.6)	0 (0)	1 (0.8)	
HAS-BLED score	1 (0, 2)	0 (0, 1)	1 (0, 2)	0.011
0	69 (43.9)	20 (62.5)	49 (39.2)	
1	55 (35.0)	9 (28.1)	46 (36.8)	
2	26 (16.6)	3 (9.4)	23 (18.4)	
3	7 (4.5)	0 (0)	7 (5.6)	
Mean of PT-INR	2.01 ± 0.27	2.02 ± 0.26	2.01 ± 0.27	0.807
TTR (%)	84 (68, 97)	83 (72, 99)	85 (67, 96)	0.595
PINRR (%)	78 (60, 90)	78 (62, 100)	78 (60, 90)	0.670
PINROR (%)	0 (0, 17)	0 (0, 15)	0 (0, 17)	0.671
PINRBR (%)	11 (0, 27)	0 (0, 28)	12 (0, 27)	0.444
CV of PT-INR	0.153 ± 0.080	0.147 ± 0.069	0.154 ± 0.083	0.632
Dose of warfarin	2.7 (2, 3.5)	3 (2.5, 3.625)	2.5 (2, 3)	0.084
Use of granule formulation of warfarin	4 (2.5)	0 (0)	4 (3.2)	0.583
Number of warfarin dose changes	0 (0, 1)	0 (0, 1)	0 (0, 1)	0.153
Medications				
Number of prescribed drugs	7 (5, 9)	3 (3, 4)	8 (6, 10)	<0.001
ARB	78 (49.7)	9 (28.1)	69 (55.2)	0.015
ACEI	28 (17.8)	1 (3.1)	27 (21.6)	0.017
Calcium channel blocker	47 (29.9)	3 (9.4)	44 (35.2)	0.004
β-blocker	111 (70.7)	19 (59.4)	92 (73.6)	0.125
Antiplatelet agent	46 (29.3)	0 (0)	46 (36.8)	<0.001
NSAIDs	5 (3.2)	0 (0)	5 (4.0)	0.584
PPI	70 (44.6)	9 (28.1)	61 (48.8)	0.069
H_2_-blocker	17 (10.8)	2 (6.3)	15 (12.0)	0.527
Statin	65 (41.4)	5 (15.6)	60 (48.0)	0.001
Oral hypoglycemic agent	26 (16.6)	2 (6.3)	24 (19.2)	0.109
Insulin	9 (5.7)	0 (0)	9 (7.2)	0.206
Antianxiety or hypnotic	21 (13.4)	2 (6.3)	19 (15.2)	0.252
Medications potentiating the action of warfarin	90 (57.3)	7 (21.9)	83 (66.4)	<0.001
Allopurinol	35 (22.3)	2 (6.3)	33 (26.4)	0.016
Rosuvastatin	23 (14.6)	2 (6.3)	21 (16.8)	0.168
Levothyroxine	14 (8.9)	2 (6.3)	12 (9.6)	0.737
Others	50 (31.8)	3 (9.4)	47 (37.6)	0.002
Medications weakening the effect of warfarin	9 (5.7)	0 (0)	9 (7.2)	0.206

The data are presented as mean ± SD for normally distributed variables, median (first quartile, third quartile) for not normally distributed variables, or the number (percentage) for categorical variables. We defined vascular disease as prior myocardial infarction, peripheral artery disease, or aortic plaque, according to CHA2DS2-VASc score (Lip et al., 2010). We also defined bleeding as bleeding history or bleeding tendency (bleeding predisposition and anemia) according to HAS-BLED score (Pisters et al., 2010) Differences in categorical variables between the NP group (number of prescribed drugs ≤4) and the PP group (number of prescribed drugs ≥5) were assessed using Fisher's exact test. Differences in continuous variables between the NP group and the PP group were assessed using unpaired Student’s *t*-test for normally distributed variables, or Mann–Whitney U-test for not normally distributed variables. ACEI, angiotensin-converting enzyme inhibitor; ARB, angiotensin receptor blocker; BMI, body mass index; Cr, serum creatinine; eGFR, estimated glomerular filtration rate; H_2_-blocker, histamine type 2 receptor blocker; Hb, hemoglobin; Ht, hematocrit; NP, non-polypharmacy; NSAID, non-steroidal anti-inflammatory drug; PINRBR, percentage of PT-INR determinations below range; PINROR, percentage of PT-INR determinations over range; PINRR, percentage of PT-INR determinations in range; Plt, platelet; PP, polypharmacy; PPI, proton pump inhibitor; PT-INR, international normalized ratio of prothrombin time; RBC, red blood cells; TTR, time in therapeutic range; WBC, white blood cells.

### Factors Correlated With the Number of Prescribed Drugs

In the univariate analyses, the number of the prescribed drugs was positively correlated with HAS-BLED score, CHADS2 score, CHA2DS2-VASc score, Cr, and BMI, and negatively correlated with eGFR and AST (Table 2). The TTR, PINRR, and CV of PT-INR were not significantly correlated with the number of the prescribed drugs. Factors with *p* < 0.10 in Spearman’s rank correlation coefficient were selected as independent factors in the multivariate analysis (i.e., eGFR, BMI, and AST). HAS-BLED score, CHADS2 score, and CHA2DS2-VASc score were omitted from independent variables because of collinearity. In the multivariate analysis, independent variables that were significantly correlated with the number of prescribed drugs were eGFR and BMI (Table 3).

**TABLE 2:** Correlation between the clinical factors and the number of the prescribed drugs.

Factor	ρ	*p*-value
CHADS_2_ score	0.440	<0.001
HAS-BLED score	0.429	<0.001
eGFR	−0.307	<0.001
Cr	0.303	<0.001
CHA_2_DS_2_-VASc score	0.275	<0.001
BMI	0.182	0.030
AST	−0.180	0.027
TTR	−0.097	0.234
PINRR	−0.064	0.427
CV of PT-INR	−0.042	0.599

Correlations between each variable were examined using Spearman’s rank correlation coefficient rho (*ρ*). Abbreviations as in Table 1.

**TABLE 3:** Multivariate factors associated with the number of the prescribed drugs.

Factor	β	*p*-value
eGFR	−0.065	<0.001
BMI	0.253	0.001
AST	−0.052	0.119

Factors which were independently associated with the number of the prescribed drugs were examined using linear regression analysis. Abbreviations as in Table 1.

### Factors Associated With PT-INR Stability

**FIGURE 1:**
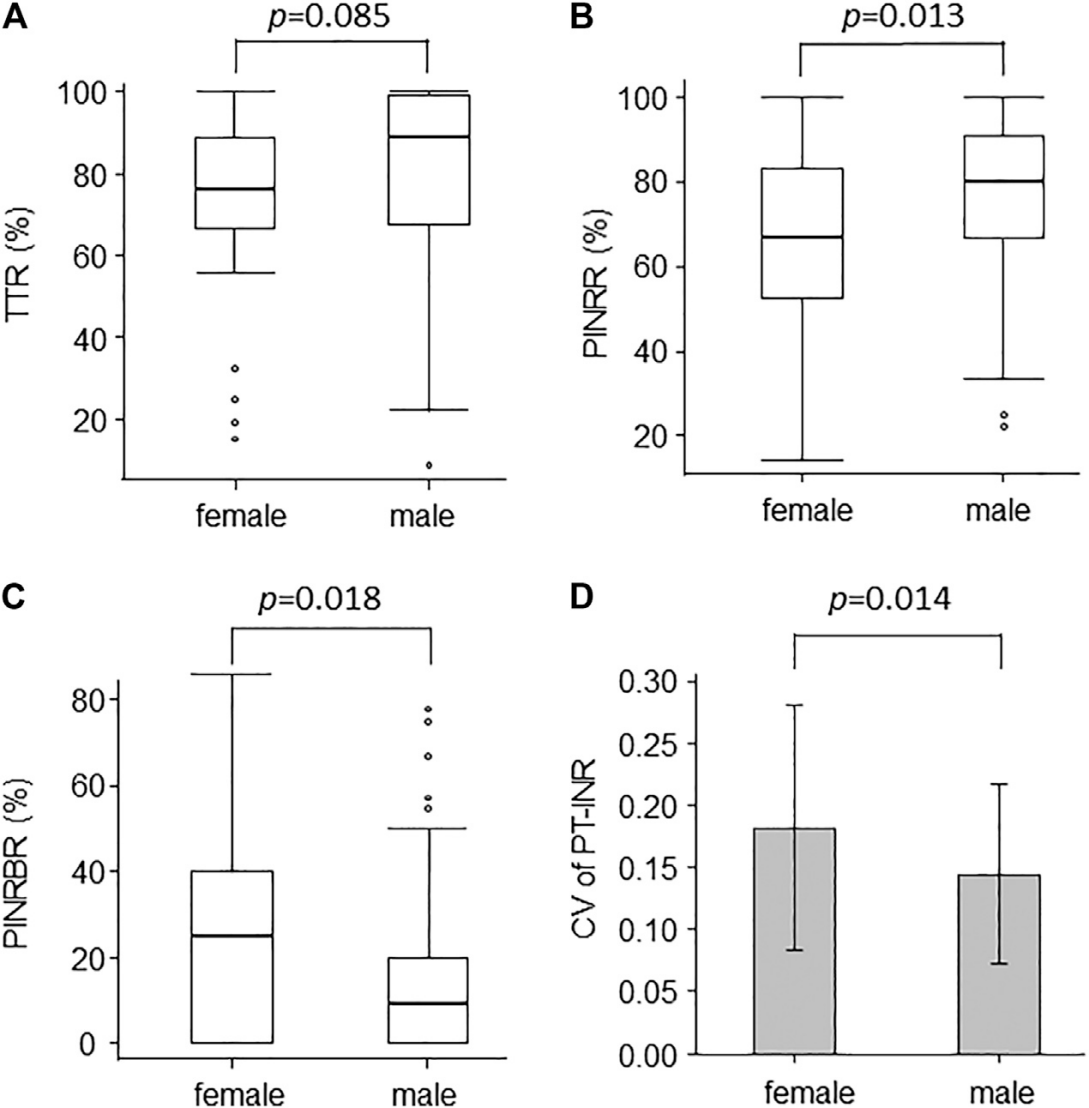
Box plots of **(A)** TTR, **(B)** PINRR, and **(C)** PINRBR show the difference between the quality of warfarin control in male and female patients. Box, interquartile range; horizontal line within box, median; whiskers, smallest and largest non-outlier values; open circles, outliers. Bar chart of **(D)** shows the CV of PT-INR in male and female patients (mean ± standard deviation). TTR, time in therapeutic range; PINRR, percentage of PT-INR determinations in range; PINRBR, percentage of PT-INR determinations below range; PT-INR, international normalized ratio of prothrombin time.

We examined factors associated with TTR and PINRR. TTR was negatively correlated with HAS-BLED score (*ρ* = −0.266, *p* < 0.001). No factors showed a positive correlation with TTR. TTR tended to be lower in female patients, but the difference was not statistically significant (Figure 1A). PINRR was positively correlated with RBC and Ht, and negatively correlated with HAS-BLED score (Table 4). PINRR was significantly lower in female patients than in male patients (Figure 1B). Factors with *p* < 0.10 in Spearman’s rank correlation coefficient or Mann-Whitney *U*-test were selected as independent factors in the multivariate analysis (i.e. gender and hemoglobin). HAS-BLED score was omitted from independent variables because poor warfarin control is one of the factors impacting HAS-BLED score. In the multivariate analysis, the independent variable that was significantly correlated with PINRR was gender (Table 5). However, there was no difference in the dose of warfarin between male and female patients (data not shown). There were no differences in CHADS2 score (*p* = 0.493, Mann-Whitney’s *U*-test), HAS-BLED score (*p* = 0.509, Mann-Whitney’s *U*-test), and the rate of polypharmacy (*p* = 0.814, Fisher’s exact test) between the female and male patients.

**TABLE 4:** Correlation with PINRR.

Factor	ρ	*p*-value
HAS-BLED score	−0.302	<0.001
RBC	0.176	0.034
Ht	0.164	0.049
Hb	0.141	0.091

Correlations between each variable were examined using Spearman’s rank correlation coefficient rho (*ρ*). Abbreviations as in Table 1.

**TABLE 5:** Multivariate factors associated with PINRR.

Factor	β	*p*-value
Male	8.978	0.033
Hb	1.123	0.281

Factors which were independently associated with PINRR were examined using linear regression analysis. Abbreviations as in Table 1.

The mean of PT-INR was significantly lower (female vs. male patients; 1.93 ± 0.26 vs. 2.03 ± 0.27, *p* = 0.035) and the percentage of PT-INR determinations below the range was higher in female patients than in male patients (Figure 1C). These findings suggested that female patients tended to be undertreated.

Next, we examined factors that were associated with the CV of PT-INR. The CV of PT-INR was larger in female patients than in male patients (Figure 1D). The rate of patients who received a change in warfarin dose tended to be higher in female patients than in male patients (female vs. male patients; 55.6 vs. 37.2%, *p* = 0.056). Thus, the difficulty in achieving stable warfarin control was thought to be the cause of undertreatment in female patients.

## Discussion

We searched for factors that affected the quality of anticoagulant therapy with warfarin. Especially, we were interested in the impact of polypharmacy on TTR, PINRR, and the CV of PT-INR, because some previous studies suggested that polypharmacy is associated with thrombosis or bleeding in patients treated with warfarin. Focks et al. showed that polypharmacy is associated with stroke and major bleeding in patients with AF (Focks et al., 2016). However, they did not determine the quality of warfarin therapy in their population. Thus, we examined whether polypharmacy deteriorated the quality of anticoagulant therapy with warfarin. In our study, polypharmacy did not affect the stability of warfarin therapy. The stability of warfarin therapy was also not related to the number of medications that were known to affect the potency of warfarin. Our results are consistent with the substudy of the Hokusai-VTE trial. Vanassche et al. have shown that polypharmacy is associated with increased recurrence of venous thromboembolism (VTE) but not with poor TTR in VTE patients taking warfarin (Vanassche et al., 2018). The deleterious effect of polypharmacy on the quality of warfarin therapy was not observed in our study as with their study, even though our study population was older and was taking a higher number of medications, which should result in poorer adherence to medication than in their study population. The quality of warfarin therapy may be resistant to poor adherence induced by polypharmacy because its effect is long-lasting. The duration of action of a single dose of racemic warfarin is 2–5 days (Bristol-Myers Squibb Company, 2011). A few incidences of forgetting to take warfarin might not induce the clinically significant variation in PT-INR. As another reason, the patients may be highly motivated for treatment and well educated about the importance of anticoagulant therapy because our study was conducted in the tertiary referral hospital. Patients who were included in a multicenter randomized controlled study may also be well motivated and educated. Therefore, polypharmacy may not deteriorate adherence in such patients. Further study is needed to determine whether this result is generalizable, especially to patients with frailty, in whom polypharmacy may have a deleterious effect on adherence.

Next, we investigated which factors affected the stability of warfarin therapy. The factors related to the stability of warfarin therapy was gender. Female gender is known to be associated with poor outcomes in patients with AF (Stewart et al., 2002; Friberg et al., 2004). However, it remains unknown why female patients experience a high incidence of stroke. Many studies have reported that TTR tend to be lower in female patients than in male patients with AF (Gomberg et al., 2006; Sullivan et al., 2012; Arbring et al., 2013; Tomita et al., 2013; Abohelaika et al., 2016; García-Sempere et al., 2019). Thus, in SAMe-TT2R2, a scoring system to assess the likelihood of poor INR control among patients with AF receiving warfarin therapy, female gender is one of the factors (Lip et al., 2016). However, to the best of our knowledge, no studies have shown why female gender is associated with the low quality of warfarin therapy. We found that female patients tended to be undertreated. The lower mean values of PT-INR and the higher percentage of PT-INR determinations below range suggested that underdosing of warfarin caused low PINRR in female patients. However, it should be considered why female patients tended to be undertreated. There were no differences in CHADS2 score, HAS-BLED score, and the rate of polypharmacy between the female and male subgroups. Thus, the risk of stroke, the risk of bleeding, and the number of concomitant diseases were similar in both gender groups and could not explain the poor INR control in female patients. We found that the CV of PT-INR was larger and the proportion of patients who received changes in warfarin dose tended to be higher in female patients than in male patients. Thus, achieving stable warfarin control appears to be more difficult in female patients than in male patients. Instability in warfarin control may result in cause undertreatment in female patients because doctors may tend to select a dose to avoid bleeding rather than thromboembolism. We tried to elucidate why warfarin control was more varied in female patients. However, we did not identify any meaningful findings. In our study population, the age was higher and body weight was lower in female patients than in male patients, but age and body weight were not associated with TTR, PINRR, and the CV of PT-INR. Further studies are necessary to elucidate the mechanisms for the poor quality of warfarin control in female patients. Anyway, we should be more careful to the quality of warfarin control in female patients with AF. Most patients in our study population were prescribed the tablet formulation of warfarin and received dose adjustment in increments of 0.5 mg. Fine tuning of warfarin dosage using granule formulation may help to improve the quality of warfarin therapy. It may be better to change from warfarin to DOACs more aggressively in female patients when economically viable.

There are several limitations in this study. First, the sample size is small. Specifically, the proportion of patient without polypharmacy is small. The proportion of female is also small. Second, the well-known factor contributing to INR instability is dietary intake of Vitamin K containing foods. This data was not available in this study.

## Conclusion

Polypharmacy did not deteriorate the quality of warfarin therapy in patients with AF treated in a tertiary referral hospital. Female gender was an independent predictor of the lower quality of warfarin therapy.

## Data Availability

The raw data supporting the conclusions of this article will be made available by the authors, without undue reservation.
